# Long-Term Opiate Therapy-Induced Secondary Adrenal Insufficiency: A Distinct Differential Diagnosis That Should Be Considered

**DOI:** 10.7759/cureus.49955

**Published:** 2023-12-05

**Authors:** Amal Mohamed Khair

**Affiliations:** 1 Internal Medicine, Jazan University, Jazan, SAU

**Keywords:** jazan, sickle cell disease, oiai, secondary adrenal insufficiency, opioids

## Abstract

Pain management with opioid medication is associated with several side effects. Opioid-induced adrenal insufficiency by suppression of the hypothalamic-pituitary-adrenal (HPA) axis is one of them that needs to be considered. The possible effects of opioid use on adrenal function are addressed in this case report.

This is a case of a 21-year-old female patient with sickle cell disease who started, for the last year, on extended-release morphine sulfate 45mg daily in an attempt to control the severity of her pain and frequent admission with the vaso-occlusive crisis. She presented with a sepsis-like presentation and received vasopressor, empiric antibiotics, and glucocorticoid. She experienced low blood pressure and low blood glucose after weaning off of steroids. A diagnosis of secondary adrenal insufficiency was established after comprehensive reevaluation and confirmed by morning cortisol value and ACTH stimulation test. Her long-term use of opioids was considered the underlying cause of her secondary adrenal insufficiency after the exclusion of other causes and the normal pituitary gland on the brain magnetic resonance image. She received maintenance hydrocortisone. On follow-up, the patient showed effective improvement, and her adrenal function recovered after discontinuation of the morphine over the following six months.

In conclusion, OIAI is an under-recognized condition of adrenal insufficiency secondary to long-term exposure to opioids. OIAI can cause symptoms and may result in potentially life-threatening adrenal crises, but it can be managed. A direct detrimental impact on the hypothalamus and pituitary gland mostly causes the suppression of cortisol secretion by opioids. Understanding how to diagnose and treat OIAI is crucial, particularly since opioids are widely used. To determine the frequency and clinical importance of opioid-induced adrenal insufficiency and if hormone replacement therapy is necessary, more research is required.

## Introduction

Opioids are considered among the commonly prescribed potent analgesic medications. They play an essential role in pain relief for acute and chronic pain in many conditions like cancer and sickle cell disease. However, there are many concerns regarding their use, such as tolerance, addiction, drug abuse, and adverse effects [1]. One of these side effects is the effect of opioids on endocrine function as a result of inhibition of the hypothalamic-pituitary axis [2,3]. The most common of which is opioid-induced hypogonadism. Adrenal insufficiency was reported in several case reports and other small research studies [2]. Opioids function by binding to opioid receptors, specifically μ (Mu opioid receptors), δ (Delta opioid receptors), and κ receptors (Kappa opioids receptors). These G protein-coupled receptors were distributed on the hypothalamus and pituitary as well as throughout the body [3].

In patients receiving long-term opioid medications, opioid-induced adrenal insufficiency (OIAI) can occur in 9% to 29% of cases [1]. In a recent "meta-analysis, de Vries et al." estimated the prevalence of OIAI to be 15% based on 205 individuals who used different opioid treatments across five research studies [4].

OIAI is difficult and creates some challenges for its diagnosis. Nausea, vomiting, tiredness, loss of weight, dizziness, and muscle aches are some of the symptoms related to OIAI. Since some of these general symptoms may coincide with the patient's original complaints, assessing them may be challenging [1,2]. OIAI is distinct from primary adrenal insufficiency in several aspects and arises as a result of extended exposure to opioids, and risk factors related to OIAI are still not clear [1,5].

Both acute and chronic opioid use can have substantial effects on the hypothalamic-pituitary-adrenal (HPA) axis [6], with possible adverse clinical consequences if treatment is not received. Opioids suppress the hypothalamus's secretion of corticotrophin-releasing hormone, which stops the anterior pituitary gland from producing ACTH [6]. When the opioid medication is stopped, the HPA axis may recover.

In this case of secondary adrenal insufficiency following long-term treatment with morphine, the main objective is to highlight the effect of opioid use for non-cancer pain on adrenal function.

## Case presentation

A 21-year-old Saudi female was brought to the hospital due to acute severe pain on both sides of her arms and legs and fever. She was diagnosed with sickle cell disease hemoglobin SS (HbSS) at the age of 10. Since then, the patient has experienced more than 20 sickle cell attacks annually. During these episodes, she was hospitalized for approximately two to three weeks and treated with intravenous (IV) fluids and opioids, and sometimes with antibiotics as required for infection. She takes 5mg of folic acid and 500mg of deferasirox daily. She discontinued using hydroxyurea due to the intolerance. She denied any glucocorticoid use.

One year ago, her treating physician began her on a regimen of 45 mg of extended-release morphine sulfate daily due to the severity of her pain and illness. After this, she stated that the treatment shortened her hospital stay and controlled her pain. During that period, follow-up was done for drug intoxication, and regular evaluation of the cardiopulmonary, eye, and gastrointestinal systems was unremarkable.

She presented, during this recent visit, with a fever of 38.1°C, tachycardia of 128/min, dizziness, headache, abdominal pain, nausea, and vomiting. In addition, she mentioned that she had amenorrhea for the last two months. Physical examination on admission revealed a blood pressure (BP) of 84/40. Respiratory rate was 20 breaths per minute, with 97% oxygen saturation on room air. Her heart had a normal rhythm, her lungs were clear upon auscultation, and her abdomen was soft and not tender. There was tenderness in all four upper and lower limbs.

Complete blood count (CBC) showed Hb of 8.0g/dL, neutrophil leukocytosis of 21,000 cells/mcl, and platelet count of 475,000. Blood glucose 69 mg/dl, liver function test (LFT), renal function test (RFT), and electrolytes were normal. A chest x-ray was unremarkable, and a normal ultrasound of the abdomen and pelvis.

The patient received aggressive IV fluid resuscitation, antiemetic and analgesic. Despite that, she continues to have vomiting and hypotension. Therefore, she was initiated on inotrope with a norepinephrine infusion and stress-dose hydrocortisone (100mg/12 hourly). An empiric antibiotic, with meropenem 1g every 12 hours, was started after a blood sample was taken for blood culture, and urine for culture was requested.

Blood and urine cultures returned negative, and a culture-negative sepsis diagnosis was made. The patient's condition improved two days later, and the fever subsided.

On day seven of the hospital course, following the discontinuation of steroid therapy, she developed postural hypotension and vomiting. Additional investigations were unremarkable, including abdominal and pelvic computed tomography and echocardiography. A further comprehensive evaluation was considered. Laboratory tests showed blood glucose 60 mg/dl, normal potassium 4.6, and normal serum creatinine and blood urea nitrogen (BUN). Test for tuberculosis and hepatitis were negative (Table 1).

**Table 1:** Initial laboratory investigations Hb: Hemoglobin; WBCS: white blood cell; BUN:  blood urea nitrogen: LDH: lactate dehydrogenase; hCG: human chorionic gonadotropin; ANA: antinuclear antibodies

Investigation	Patient results	Reference range
Hb	8.0 g/dl	12-16g/dl
WBCS	21.2 × 10^3^	4.5 – 11.0 × 10^3^/μL
Platelets	475	150 - 450
Serum Sodium	139mmol/L	135 – 145mmol/L
Serum Potassium	4.2mmol/L	3.5 -5.3mmol/L
Serum Calcium	8.9mg/dl	8.8 – 10.3mg/dl
BUN	7.4mg/dl	7 -20 mg/dl
Serum Creatinine	0.6mg/dl	0.7 -1.3mg/dl
Blood Glucose	69mg/dl	60 -115mg/dl
LDH	417U/L	140 -280U/L
Alanine transaminase	23.6U/L	10 -40
Aspartate transaminase	15U/L	10-39U/L
Albumin	3.2g/dl	2.4-4g/dl
Indirect bilirubin	1.5mg/dl	0.2 –0.8mg/dl
C-reactive protein	10mg/dl	< 0.3mg/dl
Hepatitis B surface Ag	Negative	
Hepatitis C Ab	Negative	
HCG (urine)	Negative	
Rheumatoid factor	<12UI/ml	<12UI/ml
ANA	<1:40	>1:60
Blood culture	No growth	
Urine culture	No growth	

With the persistence of symptoms and postural hypotension documented, an endocrine consultation was done, and a workup for adrenal insufficiency was performed. An 8:00 am cortisol level was 53.6 nmol/L (cutoff value <100nmol/L), and baseline adrenocorticotropic hormone (ACTH) was 4.5pmol/L (cutoff value<12pmol/L). After withholding her steroid therapy that morning, an ACTH stimulation test demonstrated a serum cortisol level of 386 nmol/L and 412.6nnmol/L, 30 and 60 minutes, respectively, with normal potassium and sodium levels, indicating intact mineralocorticoid system. Dehydroepiandrosterone sulfate (DHEAS) was low. (Table 2) 

**Table 2:** Investigations results for evaluation of secondary adrenal insufficiency ACTH: Adrenocorticopic hormone; DHEAS: Dehydroepiandrosterone sulfate; TSH: thyroid stimulating hormone; free T4: free thyroxin: FSH: follicular stimulating hormone; LH: Luteinizing hormone

Investigation	Patient’s results	Cut-off value
8 AM serum cortisol	53.6nmol/L	<100nmol/L
ACTH	4.5pmol/L	<12pmol/L
ACTH stimulation test with 250 µg corticotrophin (CST)		
Serum cortisol (30 min)	390nmol/L	<500nmol
Serum cortisol (60 min)	412.6nmol/L	<500nmol
DHEAS	54µg/dL	65 – 380 µg/dL
TSH	0.86u/ml	0.4 -4.0 u/ml
Free T4	1.3	0.7 – 2.1ng/dl
Prolactin	8.74ng/dl	<25ng/dl
FSH	6.2 IU/L	3-10 IU/L
LH	2.6IU/L	2 -8IU/L
Estradiol	89pg/ml	27 – 161pg/ml

MRI showed a normal pituitary gland, and CT of the chest was normal. A diagnosis of secondary adrenal insufficiency was made. Once all other potential causes of secondary adrenal insufficiency were eliminated, the long-term use of opioids, specifically morphine sulfate, was identified as the underlying cause.

Treatment: The patient's symptoms disappeared, and her blood pressure returned to normal once she received intravenous hydrocortisone (100 mg every 6 hours). She was given a prescription for oral hydrocortisone (20 mg in the morning and 10 mg at night) and was discharged. She also received 15 mg of morphine sulfate twice a day. After receiving treatment for three months, her menstrual cycle was restored, and she was effectively weaned off the long-acting opioids six months later upon her request. The patient continues regular follow-ups every three months, and at 8:00 am, serum cortisol concentration was 345nmol/L (normal- 140 to 690 nmol/L), and blood glucose was 97mg/dl.

## Discussion

Opioids are potent medications that play an important role in the management of chronic pain. Opioid-associated endocrinopathies are not as well recognized as other opioid-associated adverse effects, which include constipation, lethargy, nausea, vomiting, dizziness, and pruritus [1,7].

Opiate drug prescriptions for the management of chronic pain have increased. The treating physician must understand the potential risks of prescribing long-term opioid treatment. This includes being aware of the potential impact that chronic opioid usage may have on the endocrine system [7]. This case report presented OIAI in a patient with sickle cell disease with chronic use of morphine for pain relief.

Opioids and opioid derivatives are known to affect the hypothalamic-pituitary-adrenal (HPA) axis on several levels, as demonstrated in this case [6]. Lower levels of CRH and vasopressin inhibit the pituitary's ability to secrete adrenocorticotropic hormone (ACTH) and interfere directly with the adrenal glands' ability to produce cortisol and DHEA independently or through CNS down-regulation. Serum basal cortisol and plasma ACTH can be significantly suppressed by acute oral morphine treatment [6]. As a result, low cortisol levels, decreased blood glucose, and hypotension will occur. A similar finding was reported by Rabi J of a patient with sickle cell disease, who developed opioid-induced endocrinopathies, manifested as secondary adrenal insufficiency and hypogonadism, following long-term use of opioids [2,6].

Few occurrences of adrenal crises among opioid-using individuals have been documented; the majority of them are associated with chronic use of opioids for pain relief [8-10]. Additionally, data from the literature indicates that opiates may prevent the release of adrenal androgens, such as dehydroepiandrosterone DHEAS [5,6]. Various processes at the hypothalamus level control the opioid-induced suppression of the HPA axis; evidence suggests that the δ- and κ-opiate receptors are involved in the regulation of ACTH production [11].

The lack of recognized patient or opioid therapy-specific risk factors further complicates the identification of OIAI. Several risk factors for the occurrence of opioid-induced hypogonadism have been stated, such as the usage of high-dose opioids [11,12]. It's possible that prolonged opioid use is an important risk factor for secondary adrenal insufficiency [8,12]. One research paper reported a dose-dependent effect, with a prevalence rate of 22.5% in patients who received ≥25 morphine milligrams equivalent (MME)/day [8].

An 8 am serum cortisol level, an adrenocorticotropic hormone (ACTH) concentration, and a 250 µg corticotrophin stimulation test (CST) should be used to assess patients suspected of having OIAI and 500 nmol/L usually recommended as a cutoff value for cortisol level [12]. Early identification and management of OIAI are recommended because it can cause severe morbidity [13,14]. However, OIAI's presenting symptoms are typically nebulous and diffuse, making it easier to miss the diagnosis and to identify the underlying illness as the cause than the opioid therapy [1,13].

The insulin tolerance test is considered the gold standard diagnostic test for OIAI; however, it is not always available everywhere [7]. Dehydroepiandrosterone sulfate (DHEAS) is usually assessed for unclear results from biochemical testing using cortisol and ACTH [15].

The amenorrhea in this patient can be due either to inhibition of the hypothalamic-pituitary-gonadal axis or as a result of the suppression of the HPA axis with subsequent failure to create DHEA. However, in this patient, as she had normal gonadal hormones and pregnancy was excluded, the cause of her amenorrhea was most probably due to decreased DHEAS.

Management consists of reduction or cessation of opioid use, as it permits recovery of the hypothalamic-pituitary-adrenal axis. It is unknown how long it will take for the hypothalamic-pituitary-adrenal axis to recover [16,17]. Because tapering opioid replacement therapy is linked to higher risks of relapse, it might not be possible [8,18]. OIAI symptoms are reported to improve with maintenance glucocorticoids by Gibb et al. [13]. A pilot study demonstrates that opioid-treated chronic non-cancer pain patients with compromised HPA axis responses to cold pressor tests benefit from physiologic replacement dosage hydrocortisone in terms of both pain and vitality [19].

Another factor that should be considered in this patient is the effect of sickle cell disease on the adrenal function. Ischemia and necrosis of the pituitary gland secondary to vaso-occlusion and iron overload from multiple blood transfusions that lead to pituitary infiltration are anther two major predictors of OIAI in patients with sickle cell disease [20]. However, in this patient, the pituitary MRI was normal; the patient is taking an iron chelating agent and showed good recovery of the adrenal function after cessation of the medication.

In this case, the HPA axis was negatively impacted by the long-term opiate medication. If clinical symptoms and indications are present in patients receiving 100 mg of morphine equivalency for more than a year, then opioid-induced adrenal insufficiency should be clinically considered. Treatment and diagnostic procedures should be carried out with clinical discretion. Interventional pain treatments, weaning off of opioids, transitioning to alternative opioids, and, if these methods are ineffective, providing patients with information about the risks and advantages of steroid and hormone supplements should be the next course of treatment.

## Conclusions

Patients with chronic pain who get opioid analgesia exhibit a clinically significant percentage of hypothalamic-pituitary-adrenal axis inhibition. Many patients will still need comprehensive evaluation as part of their care. Thus, it's critical that medical professionals recognize this potentially serious adverse reaction and its associated complications to prevent morbidity and death in this susceptible group of patients. In this context, studies with greater numbers of participants are needed to further identify the prevalence and possible clinical consequences of adrenal insufficiency.
